# Vaginal laser for overactive bladder syndrome

**DOI:** 10.1007/s00192-020-04319-z

**Published:** 2020-05-12

**Authors:** Ioannis Charalampous, Visha K. Tailor, Alex Digesu

**Affiliations:** 1 Medical School, University of Nicosia, Nicosia, Cyprus; 2 Department of Urogynaecology, St Marys Hospital, Imperial College NHS Trust, 4th Floor, Mary Stanford Building, London, W2 1NY UK

**Keywords:** Overactive bladder syndrome, Vaginal laser

## Abstract

Overactive bladder syndrome (OAB) is defined as urinary urgency, usually with urinary frequency and nocturia, with or without urge urinary incontinence. The prevalence of OAB in adult women ranges between 11% and 42%, is particularly common in elderly people, and can overlap with the genitourinary syndrome of menopause (GSM). There is a wide approach to the treatment of symptoms, often in a stepwise fashion, including lifestyle changes, bladder retraining and pelvic floor muscle rehabilitation, drug therapy, intra-vesical botulinum toxin injections or neuromodulation. Recently, vaginal laser therapy has been proposed as an emerging minimal invasive effective treatment option for women with OAB. We explore this further.

Overactive bladder syndrome (OAB) is defined as urinary urgency, usually with urinary frequency and nocturia, with or without urge urinary incontinence. The prevalence of OAB in adult women ranges between 11% and 42%, and is particularly common with age, overlapping with the genitourinary syndrome of menopause (GSM). There is a wide approach to the treatment of symptoms, often in a stepwise fashion, including lifestyle changes, bladder retraining and pelvic floor muscle rehabilitation, drug therapy, intra-vesical botulinum toxin injections or neuromodulation. Recently vaginal laser therapy has been proposed as an emerging minimal invasive effective treatment option for women with OAB [1–6].

Fractional microablative CO_2_ and Er:YAG laser are the two most commonly used intra-vaginal therapy methods [7–9]. Vaginal laser aims to induce collagen remodelling and neocollagenesis processes. Histopathological studies have described: an increase in proliferation of the intermediate and shedding superficial cells as well as of the underlying connective tissue; an increase in the vaginal epithelium thickness; an increase in the fibroblast growth factor and transforming growth factor beta 1 (TGF-ß1) that have been advocated as responsible for laser-induced neocollagenesis and neo-angiogenesis [3]. However, how this laser-induced tissue effect translates into an improvement of OAB symptoms has never been explained and it is currently still questionable and unknown.

Several authors to date have explored the effectiveness of vaginal lasers to treat GSM, with few studies focusing only on outcomes in women with OAB symptoms. Perino et al. carried out a pilot study treating 30 post-menopausal women with OAB symptoms with three sessions of vaginal CO_2_ fractional laser over least 30 days. At 30 days, the mean OAB-q-SF scores were reduced from 18 to 8. A total of 9 women with OAB-wet also demonstrated an improvement in the incontinence episodes [9]. The absence of a randomised or a control group, the small sample size, as well as the lack of long-term follow-up, represent the main limitations of this study.

Aguiar et al. conducted a similar study on 72 post-menopausal women randomised to receive either vaginal lubrication, vaginal oestrogen or three treatments of vaginal CO_2_ fractional laser 30–45 days apart. In this study, up to 81% of the women experienced urge urinary incontinence. Two of the 24 women undergoing vaginal laser treatment were lost to follow-up at 14 weeks. A small but statistically significant improvement in ICIQ-OAB scores was described. No adverse effects were reported during the study period [10]. The results of Aguiar et al. show a small but statistically significant difference between the intravaginal fractional CO_2_ laser and topical oestrogen groups for treating OAB symptoms related to GSM [10]. Once more the sample size was too small to draw any long-term conclusions for the efficacy of vaginal laser use for the treatment of OAB symptoms.

A similar study was carried out in 2015. In a pilot study women were treated with two sessions of Er:YAG laser treatment 4 weeks apart. A total of 60% of the cohort was satisfied with the treatment and reported an improvement in OAB symptoms at 3 months. However, the benefits were not sustained at 12 months. The authors also described a temporary improvement in sexual function. Minor side effects such as vaginal discharge or spotting lasting a few days after treatment were reported [11]. This study also had limitations such as the retrospective design, the small case series and the lack of face-to-face visit at 12 months’ follow-up that is important in our opinion in order to objectively assess the laser-induced effects on the vaginal tissues. Furthermore, the subjective questionnaires at 12 months after laser therapy do not provide adequate evidence to make a strong case for the use of vaginal laser treatment.

Finally, Okui also compared the efficacy of Er:YAG laser treatment and fesoterodine and mirabegron at treating OAB symptoms in 150 post-menopausal women. Women received once-monthly laser treatment for 3 months. In this study, all three groups reported a significant improvement in OAB Symptom Scores (OABSS) at 12 months. Only 1 patient did not respond to vaginal laser treatment. No adverse events were reported in the laser group [12]. Limitations include being a single-centre, single-operator study and the absence of a placebo arm.

In summary, the current published studies on vaginal laser therapy for women with OAB symptoms present a questionable therapeutic response. They have a small study population, are limited to single centres, with no long-term follow-up or inconsistent long-term results. Finally, and more importantly, to our knowledge, no sham randomized control studies have been carried out to date. Therefore, the current evidence supporting the use of vaginal laser treatment for OAB is still weak and limited. Although adverse events are not reported by these studies, there are reports of dyspareunia and vaginal scarring in the literature. The laser-induced risks of loss of surgical tissue planes if surgical intervention is subsequently required, as well as the possible susceptibility to infection or viruses such as the human papilloma virus, have also been reported [2, 3].

In view of the lack of robust scientific evidence, to date, vaginal laser therapy should be considered an unproven option for OAB symptoms related to GSM [8–12]. In addition, promotion of this approach for the treatment of exclusively OAB syndrome is not advised without further research investigating the efficacy and safety of this potentially long-term treatment modality. This would be best served as a well-planned, sham controlled, randomised controlled study with a long-term follow-up and robust evaluation methods.
